# Draft Genome Sequences of Three β-Lactam-Catabolizing Soil *Proteobacteria*

**DOI:** 10.1128/genomeA.00653-17

**Published:** 2017-08-10

**Authors:** Terence S. Crofts, Bin Wang, Aaron Spivak, Tara A. Gianoulis, Kevin J. Forsberg, Molly K. Gibson, Lauren A. Johnsky, Stacey M. Broomall, C. Nicole Rosenzweig, Evan W. Skowronski, Henry S. Gibbons, Morten O. A. Sommer, Gautam Dantas

**Affiliations:** a Department of Pathology and Immunology, Washington University in St. Louis, St. Louis, Missouri, USA; b Center for Genome Sciences and Systems Biology, Washington University in St. Louis, St. Louis, Missouri, USA; c Department of Genetics, Washington University in St. Louis, St. Louis, Missouri, USA; d Wyss Institute for Biologically Inspired Engineering, Harvard, Cambridge, Massachusetts, USA; e U.S. Army Edgewood Chemical Biological Center, Aberdeen Proving Ground, Maryland, USA; f OptiMetrics, Inc., Abingdon, Maryland, USA; g Novo Nordisk Foundation Center for Biosustainability, Technical University of Denmark, Lyngby, Denmark; h Department of Molecular Microbiology, Washington University in St. Louis, St. Louis, Missouri, USA; i Department of Biomedical Engineering, Washington University in St. Louis, St. Louis, Missouri, USA

## Abstract

Most antibiotics are derived from the soil, but their catabolism there, which is necessary to close the antibiotic carbon cycle, remains uncharacterized. We report the first draft genome sequences of soil *Proteobacteria* identified for subsisting solely on β-lactams as their carbon sources. The genomes encode multiple β-lactamases, although their antibiotic catabolic pathways remain enigmatic.

## GENOME ANNOUNCEMENT

Antibiotic synthesis and antibiotic resistance are both ancient and well-studied features of the soil microbiome (1). Missing from our understanding of antibiotic ecology is the ultimate environmental fate of these potential carbon sources. While antibiotic catabolism has been recognized since the discovery of the first compounds (2–4), including in multiple *Proteobacteria* species (5–10), the cellular machinery underlying these phenotypes has eluded discovery. In order to facilitate the study of this phenomenon, we have undertaken the whole-genome sequencing of three soil isolates termed ABC07 (*Pseudomonas* sp. strain PE-S1G-1), ABC08 (*Pandoraea* sp. strain PE-S2R-1), and ABC10 (*Pandoraea* sp. strain PE-S2T-3). These strains were previously described as being capable of utilizing penicillin as their sole source of carbon for growth in minimal medium (9).

Each ABC strain was inoculated into 5 ml of LB from −80°C glycerol stocks (15% in LB) and grown aerobically at room temperature. ABC strain genomic DNA was extracted from cell pellets using the Mo Bio PowerMax soil kit (catalog no. 12988-10) and dissolved in Tris-EDTA (TE) buffer. Genomes were sequenced at the Edgewood Chemical Biological Center Genomics Laboratory using a 454-GS FLX sequencer, and raw .sff files were assembled *de novo* using Newbler version 2.0.01.14 (454 Life Sciences) with the following parameters: SeedStep, 12; SeedLength, 16; MinSeedCount, 1; SeedHitLimit, 10,000; HitPositionLimit, 200; MinMatchLength, 40; MinMatchIdentity, 90; MatchIdentScore, 2; MatchDiffScore, −3; and MatchUniqThresh, 12. Each sequenced genome resulted in ca. 500,000 reads encompassing ~10^8^ bp. ABC07, ABC08, and ABC10 were assembled into 137, 38, and 25 large contigs, respectively, with contig *N*_50_ metrics of 98,867, 1,179,846, and 601,724 bp, respectively.

To identify features of the genomes potentially pertinent to antibiotic catabolism, genomes were uploaded to the online KBase server (11) for annotation using RAST (12). RAST predicted 6,594, 5,771, and 5,569 total features, and 10, 8, and 8 β-lactamases or β-lactamase-like features in the genomes of ABC07, ABC08, and ABC10, respectively. The top three functional categories identified for each strain were carbohydrates, amino acids and derivatives, cofactors, vitamins, prosthetic groups, and pigments for ABC07; carbohydrates, metabolism of aromatic compounds, and amino acids and derivatives for ABC08; and carbohydrates, metabolism of aromatic compounds, and amino acids and derivatives for ABC10.

Analysis of genomes from three antibiotic-catabolizing bacteria has revealed the presence of multiple β-lactamase genes in each organism and suggests a conserved role for the metabolism of aromatic carbon sources and amino acids. Antibiotic inactivation is confirmed in these strains phenotypically (9) and, here, by genotype, and it may represent the first step in β-lactam catabolism. The release of these genomes should significantly aid in the identification of antibiotic catabolism pathways.

### Accession number(s).

This whole-genome shotgun project has been deposited at DDBJ/ENA/GenBank under the accession numbers NGUS00000000, NGUR00000000, and NGUQ00000000 for strains ABC07, ABC08, and ABC10, respectively. The versions described in this paper are the first versions, NGUS01000000, NGUR01000000, and NGUQ01000000, respectively.

## References

[1] Crofts TS, Gasparrini AJ, Dantas G 2017 Next-generation approaches to understand and combat the antibiotic resistome. Nat Rev Microbiol 15:422–434. doi:10.1038/nrmicro.2017.28. 28392565 PMC5681478

[2] Pramer D, Starkey RL 1951 Decomposition of streptomycin. Science 113:127. doi:10.1126/science.113.2927.127. 14798402

[3] Kameda Y, Kimura Y, Toyoura E, Omori T 1961 A method for isolating bacteria capable of producing 6-aminopenicillanic acid from benzylpenillin. Nature 191:1122–1123. doi:10.1038/1911122a0. 13751020

[4] Abd-El-Malek Y, Monib M, Hazem A 1961 Chloramphenicol, a simultaneous carbon and nitrogen source for a *Streptomyes* sp. from Egyptian soil. Nature 189:775–776. doi:10.1038/189775a0. 13680938

[5] Johnsen J 1977 Utilization of benzylpenicillin as carbon, nitrogen and energy source by a *Pseudomonas fluorescens* strain. Arch Microbiol 115:271–275. doi:10.1007/BF00446452. 414683

[6] Johnsen J 1981 Presence of beta-lactamase and penicillin acylase in a *Pseudomonas* sp. utilizing benzylpenicillin as a carbon source. J Gen Appl Microbiol 27:499–503. doi:10.2323/jgam.27.499.

[7] Beckman W, Lessie TG 1979 Response of *Pseudomonas cepacia* to beta-lactam antibiotics: utilization of penicillin G as the carbon source. J Bacteriol 140:1126–1128. 533766 10.1128/jb.140.3.1126-1128.1979 PMC216765

[8] Barnhill AE, Weeks KE, Xiong N, Day TA, Carlson SA 2010 Identification of multiresistant *Salmonella* isolates capable of subsisting on antibiotics. Appl Environ Microbiol 76:2678–2680. doi:10.1128/AEM.02516-09. 20173063 PMC2849224

[9] Dantas G, Sommer MOA, Oluwasegun RD, Church GM 2008 Bacteria subsisting on antibiotics. Science 320:100–103. doi:10.1126/science.1155157. 18388292

[10] Xin Z, Fengwei T, Gang W, Xiaoming L, Qiuxiang Z, Hao Z, Wei C 2012 Isolation, identification and characterization of human intestinal bacteria with the ability to utilize chloramphenicol as the sole source of carbon and energy. FEMS Microbiol Ecol 82:703–712. doi:10.1111/j.1574-6941.2012.01440.x. 22757630

[11] Arkin AP, Stevens RL, Cottingham RW, Maslov S, Henry CS, Dehal P, Ware D, Perez F, Harris NL, Canon S, Sneddon MW, Henderson ML, Riehl WJ, Gunter D, Murphy-Olson D, Chan S, Kamimura RT, Brettin TS, Meyer F, Chivian D, Weston DJ, Glass EM, Davison BH, Kumari S, Allen BH, Baumohl J, Best AA, Bowen B, Brenner SE, Bun CC, Chandonia J-M, Chia J-M, Colasanti R, Conrad N, Davis JJ, DeJongh M, Devoid S, Dietrich E, Drake MM, Dubchak I, Edirisinghe JN, Fang G, Faria JP, Frybarger PM, Gerlach W, Gerstein M, Gurtowski J, Haun HL, He F, Jain R, et al. 2016 The DOE Systems Biology KnowledgeBase (KBase). bioRxiv. doi:10.1101/096354.

[12] Overbeek R, Olson R, Pusch GD, Olsen GJ, Davis JJ, Disz T, Edwards RA, Gerdes S, Parrello B, Shukla M, Vonstein V, Wattam AR, Xia F, Stevens R 2014 The SEED and the Rapid Annotation of microbial genomes using Subsystems Technology (RAST). Nucleic Acids Res 42:D206–D214. doi:10.1093/nar/gkt1226. 24293654 PMC3965101

